# Multivalent binding of the tardigrade Dsup protein to chromatin promotes yeast survival and longevity upon exposure to oxidative damage

**DOI:** 10.21203/rs.3.rs-3182883/v1

**Published:** 2023-07-28

**Authors:** Rhiannon Aguilar, Laiba Khan, Nina Arslanovic, Kaylah Birmingham, Kritika Kasliwal, Spike Posnikoff, Ujani Chakraborty, Allison R. Hickman, Rachel Watson, Ryan J. Ezell, Hannah E. Willis, Martis W. Cowles, Richard Garner, Abraham Shim, Ignacio Gutierrez, Matthew R. Marunde, Michael-Christopher Keogh, Jessica K. Tyler

**Affiliations:** 1 Weill Cornell Medicine, Department of Pathology and Laboratory Medicine, New York, NY 10065, USA; 2 Weill Cornell/Rockefeller/Sloan-Kettering Tri-Institutional MD-PhD Program, New York, NY 10065, USA; 3 EpiCypher Inc., Durham, NC 27709, USA; 4 Weill Cornell Medicine, Pharmacology Graduate Program, New York, NY 10065 United States.; 5 Weill Cornell Medicine, Biochemistry, Cellular, and Molecular Biology Graduate Program, New York, NY 10065, USA.

## Abstract

Tardigrades are remarkable in their ability to survive extreme environments. The damage suppressor (Dsup) protein is thought responsible for their extreme resistance to reactive oxygen species (ROS) generated by irradiation. Here we show that expression of *Ramazzottius varieornatus* Dsup in *Saccharomyces cerevisiae* reduces oxidative DNA damage and extends the lifespan of budding yeast exposed to chronic oxidative genotoxicity. This protection from ROS requires either the Dsup HMGN-like domain or sequences C-terminal to same. Dsup associates with no apparent bias across the yeast genome, using multiple modes of nucleosome binding; the HMGN-like region interacts with both the H2A/H2B acidic patch and H3/H4 histone tails, while the C-terminal region binds DNA. These findings give precedent for engineering an organism by physically shielding its genome to promote survival and longevity in the face of oxidative damage.

## INTRODUCTION

Tardigrades (also termed water bears) are an invertebrate phylum of > 1,200 species with broad-reaching habitats. Many can survive desiccation, extreme temperatures, high pressure, severe irradiation, and exposure to space^1^. The mechanisms by which tardigrade species resist such extreme stressors are poorly understood. *Ramazzottius varieornatus* is highly resistant to ionizing radiation (IR); capable of surviving > 48 hours after a dose of 4000 Gy^2^, compared to the human LD50 of ~ 4.5 Gy^3^. The *R. varieornatus* Dsup (Damage suppressor) protein is chromatin associated and predicted to promote IR resistance, being absent from IR sensitive tardigrade species^4^. Indeed, when expressed in human cells, Dsup localizes to nuclear DNA and confers IR-resistance accompanied by reduced levels of single- and double-strand DNA breaks (SSBs and DSBs)^4^; it also confers protection from radiation when expressed in tobacco plants^5^.

While IR can directly induce SSBs and DSBs, much of its genotoxicity is mediated by hydroxyl radicals (OH^•^), the most powerful oxidant among the reactive oxygen species (ROS) and generated when radiation interacts with water molecules^6^. Consistent with Dsup protecting against hydroxyl radicals, it also reduces the number of DNA breaks in human cells exposed to hydrogen peroxide (H2O2)^4,7^. The high-energy hydroxyl radicals react with DNA bases to form lesions (including 8-oxoguanine; 8-oxo-G), while oxidation of the deoxyribose backbone dissociates sugar-phosphate bonds leading to DNA breaks^8^. Throughout life, oxidative DNA damage is generated from aerobic metabolism, with the resulting mutations thought to contribute to the ageing process^9^ and the development of age-related diseases^10^, such as neurodegeneration^11^ and cancer^12,13^. Most cancer treatments cause oxidative DNA damage and strand breaks, and thus contributes to long-term side effects in survivors^14^. As such, the means by which proteins such as *R. varieornatus* Dsup protect the genome from oxidative damage are of extreme interest.

*R. varieornatus* Dsup is a 445 amino acid protein predicted to be intrinsically disordered^15^. Of note, disorder at the N- and C-termini is an important feature of proteins that scan and engage DNA, consistent with a DNA-binding role for Dsup^16,17^. C-terminal deletion (Δ aa 208–445) abrogates Dsup binding to naked DNA or human chromatin^4^. Indeed, Dsup binds with higher affinity to reconstituted chromatin over free DNA, and sequences within aa 360–445 are required for the association with chromatin and protection from DSBs caused by hydroxyl radicals^18^. While Dsup induction in human cells upregulates the expression of DNA repair genes^7^; the protein also physically prevents DNA damage via chromatin binding, as this ability is observed in a reconstituted system lacking DNA repair factors^18^.

Within the Dsup C-terminal region, an eight amino acid stretch (aa 363–370, RRSSRLTS) has homology to the core consensus (RSRARLSA) of the nucleosome binding domain of vertebrate High Mobility Group-N (HMGN) proteins^18,19,20^. The chromatin binding of HMGN proteins influences a wide variety of functions (including embryogenesis, development and disease protection) across diverse cell types and species^21^. Mutation of the Dsup HMGN-like domain or deletion of its entire C-terminus respectively reduces or ablates binding to reconstituted chromatin and DNA protection from hydroxyl radicals^18^. As such, in the prevailing model revealed by use of the reconstituted system, Dsup protects the genome from DNA damage by physically shielding chromatin from hydroxyl radicals, involving the Dsup HMGN-like domain within its C-terminal sequences^18^. Whether the Dsup HMGN-like domain functions *in vivo* to mediate the interaction with chromatin and protect it from oxidative DNA damage is unknown.

Here, we show that when highly expressed in budding yeast *R. varieornatus* Dsup uses its HMGN-like domain and an additional region in the adjacent C-terminal sequences to bind chromatin and protect the genome from oxidative DNA damage in a manner dependent on chromatin engagement but independent of scavenging hydroxyl radicals. Dsup expression also extends yeast replicative lifespan in the face of chronic endogenous oxidative DNA damage. A detailed analysis of [Dsup : nucleosome] engagement finds that its HMGN-like domain mediates interaction with both the H2A/H2B acidic patch on the nucleosome surface and the H3/H4 N-terminal tails, while the distal C-terminal sequences binds DNA. Of note such a binding mechanism supports a broad engagement with *in vivo* chromatin independent of the landscape of histone post-translational modifications (PTMs). Our studies indicate that tardigrade Dsup can be introduced to a heterologous *in vivo* system and confer viability and longevity. This is achieved by physically coating the chromatinized genome via multivalent interactions to prevent hydroxyl radicals from damaging genomic DNA.

## RESULTS

### Heterologous expression of R. *varieornatus* Dsup in budding yeast protects against oxidative damage and promotes longevity in the face of increased oxidative stress

**Fig. 1.**
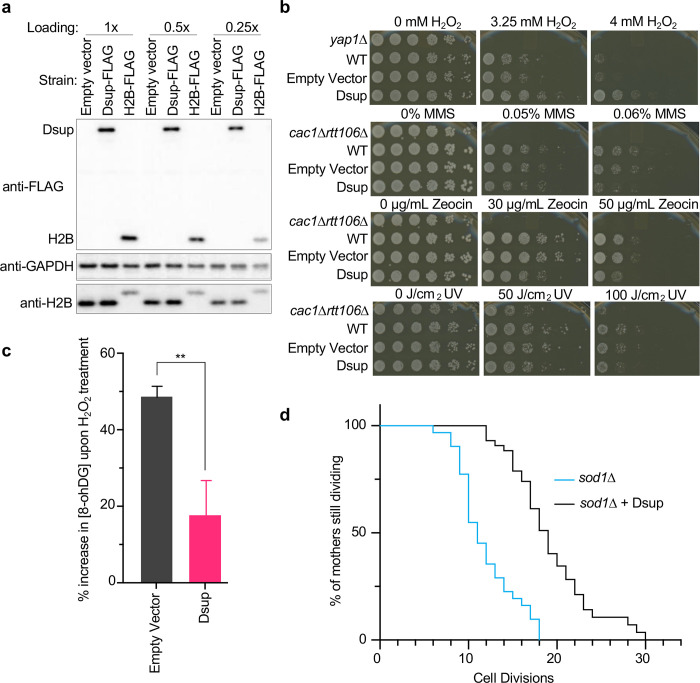
Heterologous expression of tardigrade Dsup in budding yeast promotes survival after chronic exposure to an oxidative DNA damaging agent, reduces related DNA damage, and extends lifespan upon chronic endogenous oxidative damage. **(a).** Comparative immunoblot (three dilutions of protein extracts loaded as indicated) of Dsup-FLAG (from pTDH3–6His-Dsup-FLAG) and H2B-FLAG in yeast strains containing a single integrated copy of each tagged gene (**Methods** and **Suppl. Table 2**). **(b).** Five-fold serial dilutions of strains plated on indicated concentrations of hydrogen peroxide (H_2_O_2_), methyl methanesulfonate (MMS), Zeocin, or after exposure to UV. *yap1Δ* is a positive control for sensitivity to oxidative DNA damage (H_2_O_2_); *cac1Δrtt106Δ* is a positive control for sensitivity to other tested agents. **(c).** [8-OHdG] increase (mean and standard deviation from three independent experiments; ** = p < 0.01) in response to oxidative DNA damage (120 minutes exposure to 10 mM H_2_O_2_). **(d).** Replicative lifespan of yeast undergoing chronic oxidative damage (*sod1Δ −/+* Dsup; n=30 individuals for each background).

To initiate this study we expressed epitope tagged 6His-Dsup-FLAG (hereafter Dsup-FLAG) in yeast under the constitutive high output *TDH3* promoter^22^, with the goal of achieving *in vivo* protein levels sufficient to coat the genome. Of note this yielded Dsup-FLAG of similar abundance to H2B-FLAG (Fig. 1a). To investigate the response of Dsup-FLAG yeast to chronic oxidative damage, we performed serial dilution assays on plates containing H2O2, observing a ~25-fold increased survival relative to yeast lacking Dsup (Fig. 1b). This did not extend to general protection from genotoxic insult, since Dsup-FLAG slightly decreased yeast survival in response to non-oxidative DNA-damaging agents such as alkylating methyl methanesulfonate (MMS), radiomimetic Zeocin, or UV (Fig. 1b).

In reconstituted assays recombinant Dsup protects chromatin from DSBs caused by hydroxyl radicals^18^, so we asked if Dsup expression protected the yeast genome from oxidative DNA damage. 8-oxoguanine (8-OHdG) is generated when ROS species react with DNA^23^, so we quantified the base modification after transient exposure to H2O2 and observed a significant reduction in 8-OHdG in the presence of Dsup (Fig. 1c).

ROS and oxidative damage increase with age, and reducing oxidative damage extends the lifespan of multiple species (yeast, worms, fruit flies, mice^24^), while elevated ROS production shortens lifespan^25^. We thus asked if Dsup expression could extend yeast lifespan. In otherwise WT yeast Dsup has a negligible impact on chronological lifespan (the length of time a cell survives in a non-dividing state; **Suppl. Fig. 1a**), while the replicative lifespan (the maximum number of times a cell can divide), was slightly reduced (**Suppl. Fig. 1b**). Cells lacking the superoxide dismutase (*SOD*) genes are deficient in their ability to process both endogenous and exogenous ROS. As a result, they accumulate oxidative stress and damage, such that yeast lacking *SOD1* have a shortened replicative lifespan^26^. When expressed in *sod1Δ* yeast, Dsup significantly increased their replicative lifespan (Fig. 1d), suggesting enhanced survival and longevity in the face of chronic oxidative damage.

### The Dsup C-terminus is required for protection of yeast from oxidative damage, in a manner not involving ROS scavenging

**Fig. 2.**
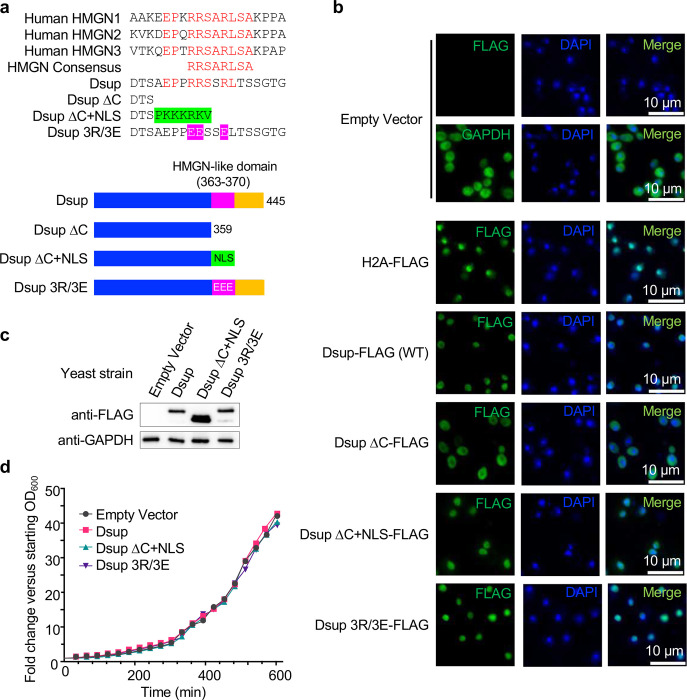
Heterologous Dsup is nuclear localized in yeast and does not negatively impact growth rate. **(a).** Alignment of the Dsup HMGN-like domain (aa 363–370, RRSSRLTS), mutants of same from this study, and the HMGN core consensus (RRSARLSA) derived from human HMGN1–3. Residues in red are identical between human HMGN1–3, the HMGN core consensus and Dsup. Green indicates the nuclear localization signal (NLS: PKKKRKVPKKKRKV) added onto the Dsup ΔC construct. Pink indicates three arginine to glutamic acid substitutions (R363E/R364E/R367E) in the HMGN-like sequence to create Dsup 3R/3E (Figure adapted from^17^). Beneath are schematics of the Dsup wild-type and mutant alleles (all containing N-terminal 6xHIS and C-terminal FLAG tags; not depicted). **(b).** Immunofluorescence to examine the subcellular location of Dsup alleles (anti-FLAG) in yeast. DAPI co-staining identifies nuclei. H2A-FLAG and GAPDH are respective controls for nuclear and cytoplasmic localization. **(c).** Western blot showing relative expression of indicated Dsup alleles (anti-FLAG) in yeast. Anti-GAPDH is a loading control for each strain protein extract. **(d).** Representative growth curves of yeast expressing Dsup alleles.

High Mobility Group-N (HMGN) proteins^18^ contain a conserved HMGN-domain (core consensus RRSARLSA^27^) required for chromatin binding and protein fuction^21^ (Fig. 2a). The Dsup C-terminal region contains an eight amino acid stretch with homology to this consensus^18^ (aa363–370, RRSSRLTS: Fig. 2a), suggesting a physiological relevance. We thus made mutant forms of Dsup by substituting three key arginines with glutamic acid within the motif (Dsup 3R/3E: R363E/R364E/R367E), or by deleting the entire C-terminus including the HMGN-like domain (Dsup ΔC: Δ360–445), alleles previously investigated *in vitro*^18^. Since Dsup contains a predicted nuclear localization signal (NLS)^28^ removed by the ΔC mutation, we added a repeated SV40 NLS (PKKKRKVPKKKRKV)^29^ C-terminal to Dsup ΔC to make Dsup ΔC+NLS (Fig. 2a). By immunofluorescence Dsup (WT), Dsup 3R/3E and Dsup ΔC+NLS localized to the nucleus, while Dsup ΔC was primarily cytoplasmic, presumably due to removal of the predicted NLS (Fig. 2b). Dsup ΔC was thus omitted from further *in vivo* study. Importantly, Dsup 3R/3E and Dsup ΔC+NLS proteins were expressed at least as well as Dsup (WT) in yeast (Fig. 2c), and the presence of each did not significantly impact cell growth (Fig. 2d).

**Fig. 3.**
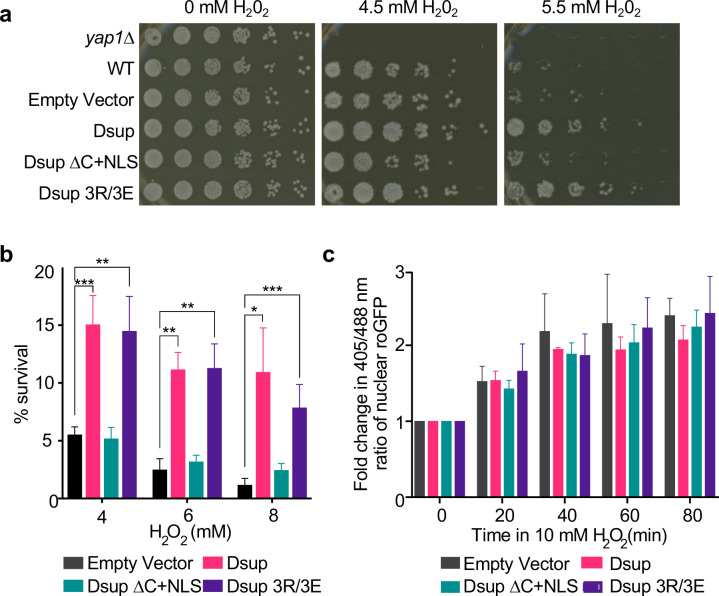
Dsup promotes survival after oxidative DNA damage in a manner that requires its chromatin binding C-terminus but is not due to ROS scavenging. **(a).** Five-fold serial dilution analysis of yeast expressing Dsup alleles plated on H_2_O_2_ (concentrations as indicated). **(b).** Cell survival after 90-minute exposure to indicated concentrations of H_2_O_2_. Shown are average and standard deviation of experiments performed from three independent yeast colonies for each strain. * = p < 0.05, ** = p < 0.01, *** = p < 0.001 by student’s t-test. **(c).** Nuclear ROS (measured by Redox analysis as in **Methods**) for indicated yeast strains. Shown are average and standard deviation of experiments performed from three independent colonies. No significant differences were observed between each time point.

We next examined the ability of Dsup mutants to enhance survival after chronic H2O2 exposure. Mutation of the HMGN-like domain (Dsup 3R/3E) protected cells comparably to Dsup (WT), while Dsup ΔC+NLS yielded no protection, with similar growth to an empty-vector strain (Fig. 3a). As such, the entire C-terminus of Dsup is important for protecting yeast against oxidative DNA damage, while the included HMGN-like domain is dispensable for this function. The observed sensitivity of yeast expressing Dsup (WT) to MMS, Zeocin and UV (Fig. 1b) was not seen upon expression of Dsup 3R/3E or Dsup ΔC+NLS (not shown), indicating that while Dsup 3R/3E can protect from oxidative damage, it does not fully mimic the WT protein.

To examine whether Dsup expression has any influence on growth following acute oxidative stress, we exposed cells in liquid culture to H2O2 for 1.5 hours, before allowing them to recover on plates with no oxidizing agent. Here Dsup or Dsup 3R/3E expression significantly (and indistinguishably) increased survival following acute oxidative stress, while Dsup ΔC+NLS conferred no protection (Fig. 3b). As such the findings from chronic and acute H2O2 exposure analyses are consistent with expression of Dsup or Dsup 3R/3E, but not Dsup ΔC+NLS, promoting yeast survival in response to oxidative stress.

Free-radical scavengers are effective at protecting yeast from oxidative stress and extending lifespan^30^. Therefore, we investigated whether Dsup acts as a free-radical scavenger. Redox-sensitive GFPs are excited at 405 nm in an oxidizing environment but 488 nm in reducing conditions, so emissions from excitation at [405/488 nm] allows the measurement of relative changes in redox state. To make a nuclear reporter for this study we added a C-terminal NLS to a roGFP2-Grx1 (glutathione reductase enzyme Grx1^31^) fusion, and confirmed the desired sub-cellular localization (**Suppl. Fig. 2**). Using this approach, we found that the redox state of the nucleus increased upon H2O2 treatment, but this was not impacted by any Dsup alleles (Fig. 3c). Therefore, Dsup expression had no influence on the yeast nucleus redox state, indicating it uses a mechanism distinct from ROS scavenging to protect the genome from oxidative damage.

### Dsup binds chromatin throughout the yeast genome, in a manner dependent on sequences within the C terminus

**Fig. 4.**
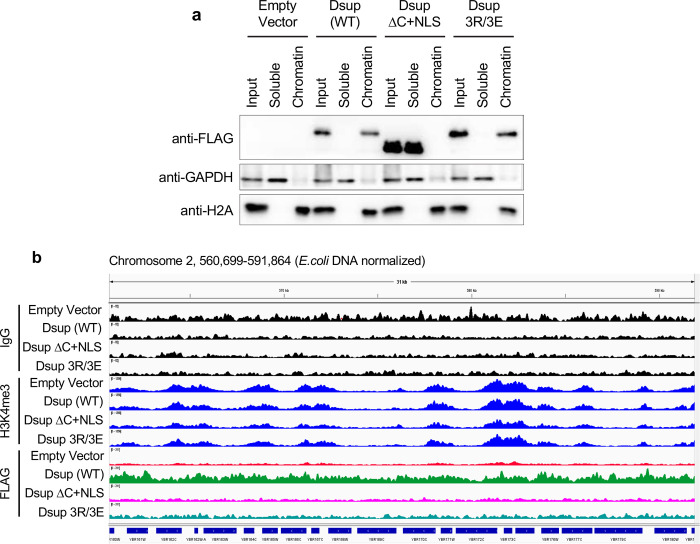
Dsup fractionates with yeast chromatin and associates across the yeast genome without apparent bias. **(a).** The Dsup C-terminus is required for stable association with chromatin *in vivo*. Total protein extracts (input) from indicated strains were separated to chromatin (H2A control) and soluble (GAPDH control) fractions and immunoblotted as indicated. **(b).** CUT&RUN analysis of Dsup allele interactions across the yeast genome *in vivo*. For each strain anti-IgG (assay background) and anti-H3K4me3 (transcriptionally active gene promoters) were respectively included as negative and positive controls. Each target is group-scaled (after normalization to *E. coli* spike-in) to the highest signal in the depicted representative IGV window (IgG (82); H3K4me3 (1356) or Dsup-FLAG (311)).

Dsup was first isolated from the chromatin fraction of Tardigrade cells^4^, and shown to bind preferentially to nucleosomes over free DNA *in vitro*^18^. Therefore, we investigated if Dsup binds yeast chromatin *in vivo*. After cellular fractionation to separate chromatin-bound from soluble proteins, Dsup and Dsup 3R/3E were enriched in the chromatin-bound fraction (Fig. 4a). By contrast, Dsup ΔC+NLS was entirely in the soluble fraction (Fig. 4a), suggesting that despite nuclear localization (Fig. 2b), it does not bind chromatin. Of note, the chromatin localization of Dsup and Dsup 3R/3E, but not Dsup ΔC+NLS, parallels their ability to promote cell survival in the face of oxidative damage (Fig. 3a,b), suggesting that chromatin binding is key.

Tardigrade Dsup expression in human and plant cells alters transcription factor binding and gene expression in response to DNA damage^5,7^. This suggests that Dsup may bind preferentially to certain areas of the genome to influence gene expression. Alternatively, to have the largest physically protective effect from oxidative DNA damage, Dsup might uniformly coat the genome. To investigate these possibilities, we used Cleavage Under Targets & Release Using Nuclease (CUT&RUN)^32^ to map 6His-Dsup-FLAG localization (by anti-FLAG) across the yeast genome, and observed that Dsup (WT) associated with all regions, with little noticeable bias or selectivity (*i.e.* without forming peaks / domains; Fig. 4b). Of note, the ability of CUT&RUN to map transcriptionally active promoters with anti-H3K4me3 was unaffected by Dsup (compare Empty vector and Dsup (WT)), indicating a minimal impact on local chromatin structure (Fig. 4b). In agreement, on titrated MNase digestion of yeast cells we observed no significant difference in chromatin accessibility between strains −/+ Dsup expression (not shown).

We next compared CUT&RUN across Dsup alleles, first noting that the relative DNA yield post MNase digestion (prior to adapter ligation) was consistently Dsup (WT) >> Dsup 3R/3E > Dsup ΔC+NLS > Empty vector (EV) (**Suppl. Fig. 3**). This suggests Dsup 3R/3E has weaker (or higher turnover) binding relative to Dsup (WT) during the CUT&RUN steps prior to MNase activation. Their relative yield is mirrored in the CUT&RUN data, where Dsup 3R/3E showed less enrichment than Dsup (WT) across all genomic regions, while Dsup ΔC+NLS resembled empty vector (Fig. 4b; all data group scaled after normalizing to *E. coli* spike-in to allow comparisons of global changes in factor binding). It would appear CUT&RUN is a more stringent analysis of chromatin interaction (presumably due [at least in part] to the long incubation times) as compared to chromatin fractionation where Dsup (WT) and Dsup 3R/3E were indistinguishable (Fig. 4a). Taken together, these data indicate that Dsup binds without obvious bias across the genome in a manner that is dependent on its C-terminus (which includes the HMGN-like domain), while mutation within the HMGN-like domain (3R/3E) reduces chromatin binding relative to wild type Dsup, but not enough to confer loss of protection from oxidative DNA damage (Fig. 3a,b).

### Dsup binds nucleosomes via multivalent interactions with the histone tails, acidic patch, and DNA

**Fig. 5.**
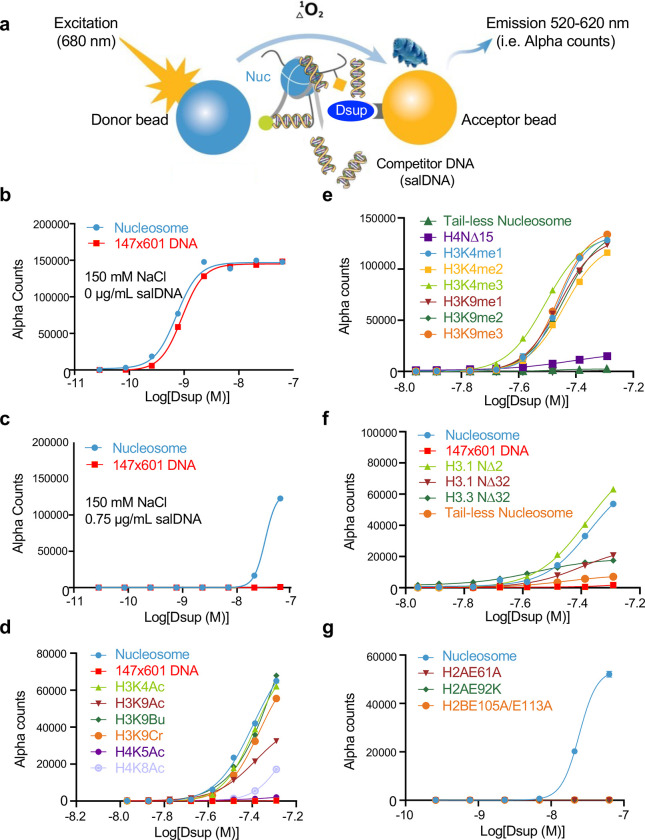
Dsup binds DNA, nucleosomal histone tails and the nucleosome acidic patch. **(a).** Schematic of the dCypher assay (**Methods**) to measure the interaction between epitope-tagged Dsup queries (**Suppl. Fig. 4**) and biotinylated nucleosome or 147×601 free DNA targets (former is depicted). The assay can be performed under a variety of conditions (*e.g.*, ionic strength) or −/+ modulators (here salmon sperm DNA (salDNA) as a competitor). **(b).** At 150mM NaCl, Dsup binds free DNA and (unmodified) nucleosome with equivalent affinities (EC_50_rel : calculated as in **Methods**^33^; see also **Suppl. Table 3** for all generated in this study). **(c).** Competitor salDNA diminishes the interaction of Dsup with nucleosomes, but ablates that with free DNA (147×601). **(d-g).** Interaction of Dsup with mononucleosomes containing defined lysine acylations **(d)**; lysine methylations **(e)**; histone tail truncations **(f)**; or acid patch mutations **(g)**. All assays performed under optimized conditions (62.5 nM Dsup (from WT), 10nM unmodified nucleosome (or 2.5 nM 147×601 (free) DNA), 150 mM NaCl, 0.75 μg/ml salDNA competitor).

To rigorously interrogate the mode of interaction of Dsup with nucleosomes or free DNA, we used the dCypher *in vitro* chemiluminescent assay^33^. Here the biotinylated target (*e.g.*, free DNA or fully defined mononucleosome) couples to streptavidin-donor beads while epitope-tagged query (here WT or mutant forms of 6His-Dsup-FLAG (**Suppl. Fig. 4**)^18^) couples to anti-tag acceptor beads. After mixing potential reactants the donor beads are excited at 680 nm, releasing a singlet oxygen that causes emission (520–620 nm) in proximal acceptor beads: this luminescent signal is directly correlated to interaction / binding affinity (Fig. 5a). To compare across each [Query : Target], data is presented as their relative concentration effective in producing 50% of the maximal response (EC50rel) by plotting Alpha Counts (fluorescence) as a function of protein concentration (see **Suppl. Table 3** for all EC50rel from this study).

To begin these studies, we titrated salt (sodium chloride) to examine the potential complication of non-specific ionic interactions (**Suppl. Fig. 5**). At lower salt (50 mM) Dsup showed slightly stronger binding to mononucleosomes over naked DNA (EC50rel 0.6 nM vs. 1.1 nM; **Suppl. Fig. 5a**), but as salt increased, the apparent affinity of Dsup for both targets gradually declined to undetectable (**Suppl. Fig. 5b-f**). We chose to move forward with approximately physiological salt (150 mM NaCl) where Dsup binding to nucleosomes and DNA was equivalent (Fig. 5b and **Suppl Fig. 5**), and next tested the impact of adding salmon sperm DNA (salDNA) as a non-specific competitor to isolate multivalent nucleosome engagement (**Suppl. Fig. 5g**)^33,34^. This identified an optimized assay condition (150 mM NaCl, 0.75 μg/ml salDNA) where nucleosome binding was retained over free-DNA (EC50rel 24.6 nM vs. Not Determined (ND); Fig. 5c); demonstrated that the Dsup-DNA association is a significant part of its interaction with chromatin; and further suggested multiple co-operative interactions between Dsup and the nucleosome.

The dCypher platform allowed us to query a diversity of fully defined mononucleosomes (**Suppl. Table 1D**) to ascertain which surfaces are most important for Dsup binding to chromatin (Fig. 5d–g). Here the apparent affinity of Dsup for mononucleosomes was minimally impacted by a variety of lysine acylations or methylations (Fig. 5d,e and not shown). However, individual deletion of the H4 N-terminal tail (NΔ15) greatly reduced the apparent affinity of Dsup (EC^50^rel 38.2 nM WT to NΔ15 ND), as did individual deletion of the H3.1 or H3.3 tails (NΔ32; both to ND), or parallel deletion of all histone tails (H2A, H2B, H3 and H4 by trypsin digestion of a nucleosome: tail-less; again to ND) (Fig. 5e,f). Of particular interest mutations (H2AE61A, H2AE92K and H2BE105A/E113A) within the nucleosome acidic patch, a hub of interaction for many nucleosome binding proteins^35,36^, profoundly impacted Dsup binding to mononucleosomes (EC^50^rel 22.8 nM WT to ND for each acid patch mutant; Fig. 5g). Taken together, these experiments indicate that Dsup interaction with chromatin is mediated by the N-terminal tails of H3 and H4, the acidic patch of H2A and H2B, and DNA.

### The Dsup HMGN-like domain mediates interactions with histones while the region C-terminal to the HMGN-like domain binds DNA

**Fig. 6.**
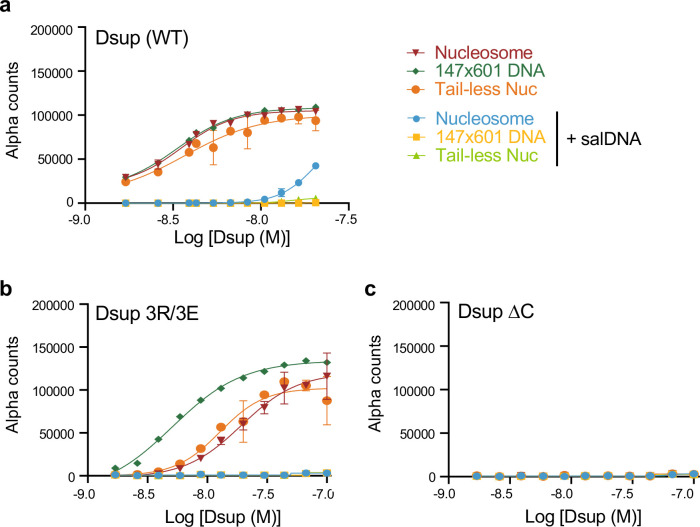
Mutation of the Dsup HMGN-like domain (3R/3E) reduces nucleosome / spares DNA binding, while C-terminal deletion (ΔC) ablates nucleosome and DNA binding. **(a-c).** Binding of Dsup alleles (WT **(a)**; 3R/E (R363E/R364E/R367E) **(b)**; or ΔC (Δ360–445) **(c)**) to nucleosome (−/+ histone tails) or free DNA (147×601) under optimized conditions (Fig. 3d–g) −/+ salDNA competitor.

We next used dCypher to examine the contribution of the Dsup HMGN-like domain and C-terminus for nucleosome and DNA engagement. In the absence of competitor DNA, 3R/3E showed noticeably reduced binding to intact and tail-less nucleosome relative to free DNA (compare to WT: Fig. 6a,b). The addition of competitor DNA (to conditions optimized for Dsup WT) then reduced nucleosome binding by Dsup 3R/3E below the level of detection (Fig. 6a,b). In profound contrast, Dsup ΔC (which lacks the HMGN-like domain and C-terminal sequences) showed no interaction with nucleosomes or free DNA under conditions optimized for Dsup (WT) (Fig. 6c), only binding at reduced NaCl (**Suppl. Fig. 6**). Such non-physiological salt concentrations facilitate nonspecific interactions between proteins and DNA^37^, so it is unlikely that the observed binding of Dsup ΔC has any functional relevance for Dsup *in vivo*. Together, these studies indicate that the Dsup interaction with nucleosomal histone tails and the acidic patch are mediated by the HMGN-like domain, while C-terminal sequences may mediate the interaction with DNA in physiological conditions.

### Dsup interaction with either the histones or DNA is sufficient to survive oxidative damage

**Fig. 7.**
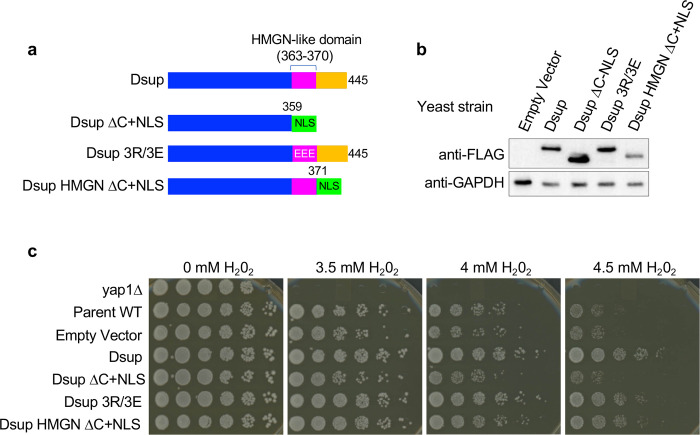
The Dsup HMGN-like domain and downstream C-terminal sequences redundantly contribute to survival during oxidative damage. **(a).** Schematic of Dsup alleles (all containing N-terminal 6xHIS and C-terminal FLAG tags (not depicted; see also Fig. 2a), including Dsup HMGN ΔC+NLS (Δ374–445 followed by an exogenous NLS (PKKKRKVPKKKRKV)). **(b).** Western blotting of expression levels (see also Fig. 2c). **(c).** Sensitivity to oxidative DNA damage (H_2_O_2_ ; see also Fig. 1b).

**Fig. 8.**
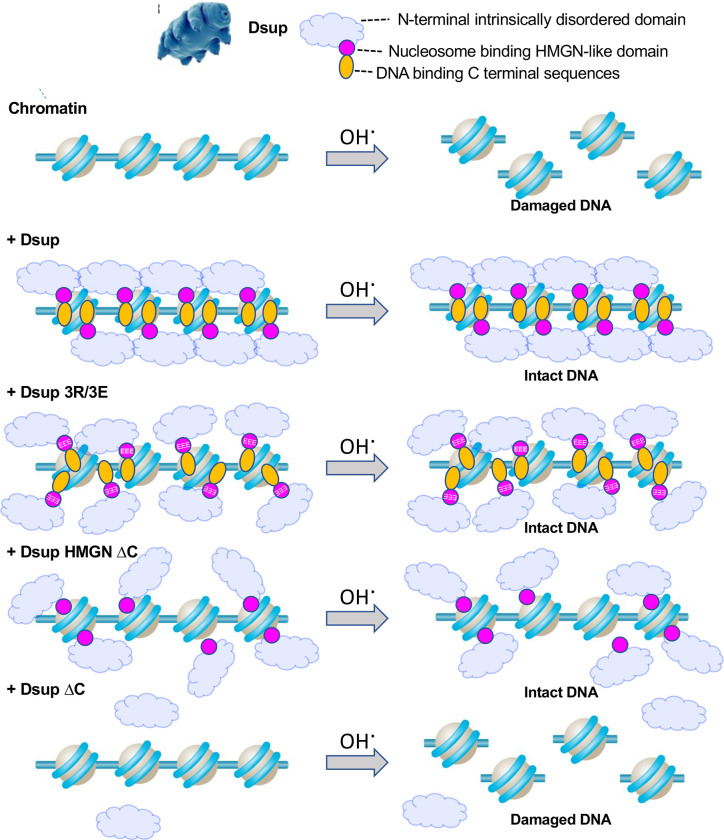
Model for multivalent association of Dsup with the genome to protect from oxidative DNA damage. Multivalent binding of Dsup to the chromatinized genome protects against oxidative DNA damage (as exogenously induced by H_2_O_2_). Dsup mutations that independently diminish interaction with the nucleosome acidic patch / histone tails (HMGN-like domain; pink), or DNA (C-terminal distal sequences; orange) have reduced chromatin interaction but are still capable of protecting the genome against H_2_O_2_-mediated DNA damage. However, loss of both interacting regions ablates the Dsup interaction with chromatin, and thus its ability to protect from oxidative DNA damage.

Our finding that the Dsup-DNA interaction remained intact after mutation of the HMGN-like domain (aa 363–370: Fig. 6b), but was lost on deletion of the entire C-terminus (Δ360–445: Fig. 6c), suggests that Dsup nucleosome and DNA interactions are mediated by distinct elements within the region previously defined as the Dsup C-terminal domain (aa 208–445)^4^. To determine the relative functional importance of these elements we created a Dsup construct that retained the HMGN-like domain, and only removed the DNA-binding C-terminus (Dsup HMGN ΔC+NLS: (Δ371–445 + NLS [PKKKRKVPKKKRKV]) (Fig. 7a). This allele is expressed in yeast at slightly reduced levels relative to the other forms of Dsup (Fig. 7b) but was notably able to promote yeast survival in the face of chronic H2O2 exposure (Fig. 7c). These data show that either an intact HMGN-like domain or intact C-terminal downstream sequences, by respectively binding to the nucleosome or DNA, are sufficient for Dsup to protect the genome against oxidative damage (Figure 8).

## DISCUSSION

To understand the molecular basis of the extreme radioresistance of tardigrades, we investigated if, and how, their Dsup protein protects against oxidative damage *in vivo*. When expressed in budding yeast, Dsup coated the entire genome without apparent bias, using two C-terminal regions to associate with chromatin via multivalent interactions involving several nucleosome surfaces and DNA. Functionally, this engagement prevents oxidative DNA damage in a manner independent of ROS scavenging. Our data supports a model where Dsup mediates multivalent interactions with chromatin to protect the underlying genome from oxidative DNA damage (Fig. 8), thus promoting yeast survival and longevity after exposure to elevated levels of hydroxyl radicals (Fig. 1b,d).

HMGN proteins are primarily described in vertebrates^38^, but whether the Tardigrade Dsup HMGN-like domain is functionally important *in vivo* was unknown. Human HMGN2 (and likely the other family members) binds nucleosomes at the H2A/H2B acidic patch^39^. Dsup also binds the acidic patch, with this interaction lost upon mutation of all three arginines in its HMGN-like domain (3R/3E; R363E/R364E/R367E: Fig. 5g). However, we additionally find that the Dsup HMGN-like domain binds histone tails, as this interaction is again lost in Dsup 3R/3E (Fig. 5e,f). Deletion of the histone tails or mutations within the H2A/H2B acidic patch are each sufficient to abolish the Dsup interaction with nucleosomes (Fig. 5e,f), suggesting its HMGN-like domain may bind these regions cooperatively. Despite the popular conception that the histone tails usually extend from the globular nucleosomal core^40^, recent work instead suggests their default high affinity interaction is with nucleosomal DNA^41^, which could potentially place the tails close to the nucleosomal acidic patch to facilitate interactions of both entities with a single HMGN-like domain. Alternatively, the HMGN-like domains of different Dsup protein molecules may bind to the histone tails and the acidic patch of the nucleosome.

Dsup is predicted to be intrinsically disordered^15^, which may allow it to wrap multiple features of nucleosome surfaces, shielding DNA from damage. The region of Dsup spanning the HMGN-like nucleosome binding domain is negatively charged and could facilitate interactions with positively charged histone tails. Meanwhile, the Dsup C-terminus is enriched in positively charged amino acids, which would facilitate ionic interactions with negatively charged DNA^15^. These interactions with histones and DNA are likely to cooperatively recruit Dsup to chromatin, and further promote non-specific coating of multiple surfaces. We observe a functional redundancy in the various Dsup interactions with chromatin *in vivo*, since individually disrupting the HMGN-like domain (Dsup 3R/3E) or adjacent C-terminal region (Dsup HMGN ΔC), that respectively compromised interactions with the nucleosome acidic patch / histone tails or DNA, still improved yeast survival on exposure to oxidative damage (Fig. 7c). Functional redundancy in the multiple interactions Dsup makes with chromatin may facilitate its recruitment even if certain surfaces are blocked by other chromatin / DNA binding proteins, potentiating its ability to coat the genome.

The observation that Dsup binding to the nucleosome was largely agnostic to most histone PTMs *in vitro* (Fig. 5d,e) is consistent with our finding that Dsup covers the entire *in vivo* genome rather than being enriched / excluded from certain regions with their particular histone modifications (Fig. 4b). Importantly, *Ramazzottius varieomatus* histones are highly conserved with human histones (as used in the dCypher assay) (**Suppl. Table 1E**), while yeast and human histones are even more similar. As a result, we consider that the observed interactions between Dsup and human or yeast histones are relevant for how the protein helps to protect tardigrades from irradiation.

In initial testing we expressed Dsup from a range of yeast promoters of various strengths, but only the very strong *TDH3* promoter enabled protection from oxidative damage (not shown and Fig. 1b). Of note, this Dsup expression level was equivalent to that of histone H2B (Fig. 1a), suggesting Dsup may be in sufficient abundance for at least two molecules per yeast nucleosome. Given that highly expressed Dsup protects the genome from oxidative DNA damage (Fig. 1c), is bound to chromatin genome-wide (Fig. 4a,b), and redundantly interacts with multiple nucleosome surfaces (Fig. 5), it is likely that Dsup non-specifically coats the *in vivo* genome to physically protect from oxidative damage, as was proposed from the previous *in vitro* studies^18^. It may be relevant to note that when we yeast-codon optimized the tardigrade Dsup protein in an attempt to promote still higher expression levels, the resulting yeast were inviable, suggesting that too much Dsup is deleterious. In agreement, expression of codon-optimized Dsup in cultured rat neurons, had detrimental effects^42^.

There is precedent for proteins binding to the genome to provide protection from irradiation and H2O2. Previous studies have demonstrated that chromatin compaction protects DNA from free radical-mediated damage caused by ionizing radiation or iron^43–45^. These findings are consistent *in vivo* and *in vitro*, suggesting a direct protection of DNA from damage rather than a particular feature of the cellular environment. Compacted chromatin also provides protection from ROS damage after direct incubation with H2O2^46^. Additionally, the deletion of proteins involved in chromatin assembly and disassembly, including the remodelers ISWI, Chd1, and INO80, renders chromatin more sensitive to DNA damage^47^.

It is intriguing that Dsup expression protected yeast from oxidative damage, but not from MMS, bleomycin, or UV: indeed, it actually increased sensitivity to these agents (Fig. 1b). Future studies should examine whether there is delayed repair of the DNA lesions generated by these genotoxins, potentially due to Dsup hindering access of the repair machinery. We note, however, that the growth rate of Dsup yeast was not reduced (Fig. 2d), indicating they are fully capable of transcriptional regulation, DNA replication and mitosis - other events one could imagine might also be prone to complications from the genome being coated with Dsup protein - but that did not appear to be the case.

These findings provide precedent for the development of organisms that can survive and live longer in the face of oxidative damage, potentially expanding the range of applications for developing therapeutic interventions by biotechnology, and furthering efforts towards human resistance to extraterrestrial effects.

## MATERIALS AND METHODS

### Yeast strains, primers, and plasmids

The pRS306-PTDH3-Dsup plasmid was created by Gibson cloning (NEB Gibson Assembly^®^ Cloning Kit) as follows. Plasmid pRS306^48^ was digested with SacI and BglII. The *TDH3* promoter was PCR amplified (primer sequences in **Suppl. Table 1A**) from yeast genomic DNA with forward primer pTDH3_SacI_F (giving homology to Sac1 digested end of pRS306), and reverse primer pTDH3_R (giving homology to 5’ end of Dsup gene). The tarigrade (*Ramazzottius varieornatus*) Dsup gene (aa1–445; encoding protein accession P0DOW4, **Suppl. Table 1E**) including an N-terminal 6xHis and C-terminal FLAG tag was amplified from plasmid pET21b-nHis6-Rvar-DSUP-cFLAG (kind gift from James Kadonaga^18^), with primers Rvar_Dsup_F and Rvar_Dsup_R, respectively giving homology to the 3’ end of the *TDH3* promoter and the 5’ end of the *ADH1* terminator. The *ADH1* terminator was amplified from yeast genomic DNA using primers tADH1_F and tADH1_BglII_R, respectively giving homology to the 3’ end of the Dsup gene and the BglII digested end of pRS306. Gibson cloning of amplified DNA fragments was carried out following kit directions.

Plasmid pRS306-PTDH3-Dsup was digested with MfeI and integrated into yeast strain BY4741 ^49^ at site of the endogenous *TDH3* promoter to make *Dsup* strain, RGY002 (pTDH3–6His_Dsup_FLAG: full list of yeast strains and their phenotypes in **Suppl. Table 1B**). Further mutations of the Dsup gene, including deletion of the C-terminus to derive *Dsup ΔC* (Δ359–445; RAY136), addition of an NLS (PKKKRKVPKKKRKV) to derive *Dsup ΔC+NLS* (RAY228), glutamic acid substitution of three arginines in the HMGN-like sequence (R363E/R364E/R367E) to derive *Dsup 3R/3E* (RAY153), and insertion of a stop codon (at Dsup codon 2) to derive *Empty vector* strain (RAY149), were made after integration using CRISPR-Cas9 mediated genome editing^50^. *Dsup HMGN ΔC+*NLS (Δ374–460, RAY274) was derived from *Dsup ΔC+NLS* by reintroduction of the HMGN consensus sequence region (aa360–373). Primer sequences used to generate guide RNAs and HDR template DNA are in **Suppl. Table 1A**.

Plasmid p415TEF cyto roGFP2-Grx1-NLS was made from p415TEF cyto roGFP2-Grx1 (kind gift from Tobias Dick; Addgene plasmid # 65004)^31^ by traditional cloning. First, the roGFP2-Grx1 sequence was PCR-amplified from plasmid p415TEF cyto roGFP2-Grx1 using primers that added a 2xNLS sequence (PKKKRKVPKKKRKV) to the Grx1 C-terminus. The resulting PCR product was digested with BamHI and HindIII and ligated to similarly digested plasmid p415TEF cyto roGFP2-Grx1.

Yeast culture and handling was performed using standard methods. Growth of strains expressing Dsup was in SC-ura media (unless otherwise indicated). All strains were isogenic to BY4741^49^ (**Suppl. Table 1B**).

### Immunoblot analysis

~10^7^ exponentially growing yeast cells (OD_λ_600 0.8–1.0) were collected by centrifugation, washed once with water, and flash frozen in liquid nitrogen before being resuspended in 100 μL modified Laemmli buffer ^51^ and boiled for five minutes. Proteins were resolved by 10% SDS-PAGE, membrane transferred, and immunoblotted with antibodies to FLAG (Sigma F1804, 1:1,000) and GAPDH (Sigma A9521, 1:10,000).

### Growth curve analysis

Yeast were grown to saturation overnight in YPD at 30°C and diluted to OD_λ_600 0.1–0.2. Growth measurements (OD_λ_600) of cultures grown from three independent colonies were taken every 30 minutes and plotted over time. Growth curves were fitted with an exponential regression using Microsoft Excel, and doubling times calculated as the slope of the curve during exponential phase. Doubling times of independent growth curves were compared using a student’s t-test.

### Chromatin fractionation analysis

~4×10^8^ exponentially growing yeast cells (OD_λ_600 0.8–1.0) were collected by centrifugation, washed once with ice cold 10% glycerol, and flash frozen in liquid nitrogen. After thawing on ice, the cell pellet was washed (100 mM Tris pH 9.4, 10 mM DTT), resuspended in the same buffer, and rested on ice for 10 minutes. Cells were pelleted by centrifugation, washed in spheroplasting buffer (10 mM HEPES, 1.2 M Sorbitol, 0.5 mM PMSF), resuspended in spheroplasting buffer containing 56 μg/mL Zymolyase 100T (US Biological), and incubated at 30°C with rotation for 1 hour. Spheroplasts were collected by gentle centrifugation, washed once in spheroplasting buffer, and once in wash buffer (1 M sorbitol, 20 mM Tris pH 7.5, 20 mM KCL, 2 mM EDTA, 0.5 mM PMSF, 0.1 μM spermine, 0.25 μM spermidine, Calbiochem Protease Inhibitor Cocktail Set IV (1:100)). Cells were gently resuspended and lysed in 250 μL Lysis Buffer (wash buffer with 400 mM sorbitol) for 10 minutes on ice.

Half of the volume after lysis (Input fraction) was boiled for 5 min in 5x Laemmli buffer, while the other half was pelleted at 14,000 x g for 15 minutes (chromatin fraction). The supernatant was collected (non-chromatin fraction) and boiled for 5 minutes in 5x Laemmli buffer, and the pellet (chromatin fraction) resuspended in 1x Laemmli buffer and boiled for 5 minutes. 7.5% of the total volume of each sample was resolved by 12.5% SDS-PAGE, membrane transferred, and immunoblotted with anti-FLAG (Sigma F1804, 1:1,000) to detect Dsup. Successful fractionation was confirmed with anti-H2A (Abcam ab18255, 1:5,000) as a chromatin bound protein, and anti-GAPDH (Sigma A9521, 1:20,000) as a non-chromatin bound protein.

### Immunofluorescence analysis

Yeast indirect immunofluorescence was carried out following published methods ^52^. 2.5 OD of early-mid log phase cells (OD_λ_600 0.5–0.6) were crosslinked in 4% formaldehyde for 20 mins at room temperature, then spheroplasted in 500 μg/mL Zymolyase 100T (US Biological) for 30 minutes at 30°C with rotation. Spheroplasted cells were applied to a 10-chamber poly-lysine coated microscope slide and permeabilized by a six minute incubation in methanol at −20°C, immediately followed by a 30 second incubation in acetone at −20°C. After blocking in 5% BSA, slides were incubated with primary antibodies to H2A (Abcam ab18255, 1:1.000), GAPDH (Sigma A9521, 1:5,000), or FLAG (Sigma F1804, 1:1,000). Incubation with Alexa Fluor^®^ 594 or 488 secondary antibodies (BioLegend) followed, and coverslips were mounted using ProLong^™^ Gold Antifade Mountant with DAPI (Invitrogen). Images were taken using an Olympus BX63 Fluorescence Microscope with a DP80 Camera and 60X objective.

### Acute and chronic damage sensitivity analysis

To measure resistance to acute hydrogen peroxide (H2O2) exposure, cells were grown in liquid YPD media until mid-log, harvested by centrifugation, and resuspended to 0.6 OD in fresh media containing H2O2 (0, 4, 6, or 8 mM). After 90 minutes growth (30°C, with shaking), cultures were diluted and spread on SC-ura agar plates. After two days at 30°C, colonies were counted and averaged across three technical replicates. Three experiments were performed from separate starting colonies, and statistical analysis performed using a student’s t-test.

The response to chronic H2O2 exposure was examined using a serial dilution assay. Cells were grown in liquid culture until mid-log (OD_λ_600 0.5–1.0), harvested by centrifugation, and resuspended in sterile water to OD_λ_600 1.0. Five-fold serial dilutions were made in a 96-well plate, and yeast spotted using a sterile 6x8-prong inoculating manifold onto YPD agar plates containing indicated concentrations of H2O2. Similar methods were used to evaluate sensitivity to methyl methanesulfonate (at the indicated concentrations in YPD) and Zeocin (at the indicated concentrations in YPD). For ultraviolet light sensitivity, yeast serial dilutions were onto YPD plates and exposed to UV (at the doses (J/cm^2^) indicated in figure legends) using a crosslinker [Stratalinker]. Plates were incubated for 3 days at 30°C.

### Replicative lifespan analysis

Cells were grown overnight to early-mid (OD_λ_600 0.2–0.6) and diluted to OD 0.1 in freshly-filtered YPD. This innoculum was added to an iBiochips automated dissection chip to achieve single cell loading as per manufacturer’s instructions. Light microscopy images of cells were acquired every 20 minutes over four days using an Evos FL Auto two-cell imaging microscope and associated software (ImageJ). At least 50 cells were counted per condition, with survival curves calculated on Graphpad Prism 9, and statistical analysis performed with a log-rank test.

### Chronological lifespan analysis

Chronological lifespan was measured according to published methods^53^. Data is presented as average and standard deviation across three independent cultures, each of which is an average of two technical replicates.

### Redox analysis

Cells expressing nuclear roGFP (p415TEF roGFP2-Grx1-NLS) were grown to mid-log (OD_λ_600 0.6–0.8). Cells were diluted in SC-ura media to OD_λ_600 0.6 in 5 mL flow cytometry tubes. Fluorescence at 405 nm and 488 nm was measured on a flow cytometer (BD Biosciences BD^®^ LSR II) immediately before direct addition of H2O2 (2 mM or 10 mM). Subsequent fluorescence measurements were taken every 20 minutes over 80 minutes.

The mean of the 405/488 nm values for each timepoint was calculated using FlowJo, with the value at time 0 normalized to 1 for each strain. Data is presented as the mean and standard deviation of three independent cultures and compared using a student’s t-test.

### ELISA for 8-OHdG

30 mL yeast cultures were grown at 30°C in shaking flasks until OD_λ_600 0.6. Cells were harvested by centrifugation, and half of each culture resuspended in either 15 mL of fresh SC-ura media or that containing 10 mM H2O2. After two hours growth at 30°C, cells were again harvested by centrifugation and genomic DNA isolated (Thermo Scientific Yeast DNA extraction kit). Genomic DNA was resuspended in 50 μL of nuclease-free water and stored overnight at 4°C.

DNA concentrations were measured using a NanoDrop spectrometer, diluted in water to 2 mg/mL, boiled for five minutes at 95°C, then immediately placed on ice for 10 minutes (to denature double-stranded DNA). 50 μg of DNA (25 μL) were sequentially incubated with Nuclease P1 (NEB: 1 unit for 2 hours at 37°C in provided buffer) and alkaline phosphatase (NEB Quick CIP: 10 units for 1 hour at 37°C in provided buffer supplemented with 100 mM Tris pH 8). Samples were incubated to denature enzymes (10 minutes at 95°C), then spun at 6000 x g for 5 minutes. DNA concentrations were measured on a NanoDrop spectrometer to ensure even loading onto the ELISA plate.

ELISA to 8-hydroxy 2 deoxyguanosine was performed as per kit instructions (Abcam ab201734). 15 μg of DNA was loaded into each of three triplicate wells for each sample (with three independent cultures measured for each condition). Absorbance at 450 nm was measured using a plate reader.

### CUT&RUN analysis

Nuclei from yeast cells expressing Dsup alleles (**Suppl. Table 1B**) were purified according to published methods^54^ with slight modifications. Yeast were grown in 500 mL of SC-ura media to OD_λ_600 0.6–0.8. Cells were spheroplasted using 500 μL of 2 mg/mL Zymolyase 100T (37°C for ~ 30 mins; until a 50 μL aliquot mixed with 1 mL of 10% SDS had an OD_λ_600 ~10% of the starting value). Remainder of the nuclei isolation was performed as previously^54^, and 1 mL aliquots containing 5 × 10^7^ nuclei were slow-frozen in an isopropanol chamber at −80°C overnight.

For CUT&RUN nuclei were rapidly thawed (2–3 minutes at 37°C), and 100 μL of suspension (5 × 10^6^ nuclei) used per reaction with the CUTANA^™^ ChIC/CUT&RUN Kit (version 3.2; EpiCypher). After immunotethering (to Rabbit IgG (EpiCypher), SNAP-Certified^™^ anti-H3K4me3 (EpiCypher), or anti-FLAG (DYKDDDDK Tag; ThermoFisher MA1-91878): **Suppl. Table 1C**) MNase digestion was performed for two hours at 4°C, and DNA eluted in 12 μL final volume.

5 ng of DNA was used to prepare sequencing libraries with the Ultra II DNA Library Prep Kit (NEB #E7645L). Libraries were sequenced on an Illumina NextSeq 2000 platform, obtaining an average of ~1.1 million paired-end reads per reaction (**Suppl. Table 2**). Paired-end fastq files were aligned to the *sacCer3* reference genome using Bowtie2. Duplicate (SAMtools) and multi-aligned (Picard) reads were filtered, and the resulting unique reads for comparable reactions normalized by an *E. coli* scaling factor (1/ % E. coli Reads) (bedtools), and further normalized to RPKM bigwig files (DeepTools). Integrative Genomics Viewer (IGV) was utilized for the visualization of peaks from bigwig files. All sequencing data has been deposited in the NCBI Gene Expression Omnibus (GEO) with accession number GSE237436.

### PTM-defined nucleosomes

All mononucleosomes (EpiCypher; **Suppl. Table 1D**) were created from fully-defined (PTM or mutant) octamers wrapped by 5’ biotinylated 147×601 DNA (**Suppl. Table 1E)** unless stated otherwise, with modifications confirmed by mass-spectrometry and immunoblotting (if an antibody was available)^33,55^. Histone tail truncations were by direct expression of the indicated histone prior to octamer assembly (H3.1 NΔ2, H3.1 NΔ32, H3.3 NΔ32, or H4 NΔ15), or trypsin digestion of assembled unmodified nucleosome (tail-less).

### dCypher binding assays

dCypher assays on the Alpha (Amplified luminescence proximity homogeneous assay) platform to examine the interaction of WT or mutant 6His-Dsup-FLAG (kind gift from James Kadonaga)^18^: the Queries [**Suppl. Table 1E**]) with free DNA (147×601 Widom sequence) or fully defined nucleosomes (the Targets: **Suppl. Table 1D**) were performed as previously^33,55^ with minor modifications.

In 384-well plates, 5 μL Dsup queries were serially titrated (in duplicate) against a fixed concentration of target (10 nM biotinylated nucleosome or 2.5 nM free DNA (147x601)). After incubation (30 minutes), interactions were detected with addition of a 10 μL mix of AlphaScreen streptavidin Donor (Revvity, 6760002) and nickel-chelate Acceptor beads (Revvity, AL108M). Following a final incubation (60 minutes), Alpha counts were measured using a PerkinElmer 2104 EnVision plate reader (680 nm laser excitation, 570 nm emission filter ± 50 nm bandwidth). Experiments were performed to assess [Query : Target] binding over a range of assay conditions (20 mM Tris pH 7.5, 0.01% BSA, 0.01% NP-40, 1 mM DTT with additives as noted), including the impact of ionic strength (50 – 250 mM NaCl) and competitor salmon sperm DNA (salDNA; 0 – 20 μg/mL). All incubations were performed at room temperature in subdued lighting. Binding curves were plotted in GraphPad Prism 9.0 using 4-parameter logistic nonlinear regression.

Binding curves [Query : Target] were generated using a non-linear 4PL curve fit in Prism 9.0 (GraphPad) to yield EC50rel values^33,55^ (**Suppl. Table 3**). Where necessary, values beyond the Alpha hook point (indicating bead saturation / competition with unbound Query) were excluded and top signal constrained to average max signal for Target. In cases where signal never reached plateau, those were constrained to the average max signal within the assay (relative to unmodified nucleosome). In remaining cases, when a targets maximal signal never achieved half of max signal relative to unmodified nucleosome, an EC50rel was deemed not determinable (ND).
